# Evolution of Hypodensity on Non-Contrast CT in Correlation with Collaterals in Anterior Circulation Stroke with Successful Endovascular Reperfusion

**DOI:** 10.3390/jcm11020446

**Published:** 2022-01-16

**Authors:** Michał Gębka, Anna Bajer-Czajkowska, Sandra Pyza, Krzysztof Safranow, Wojciech Poncyljusz, Marcin Sawicki

**Affiliations:** 1 Department of Diagnostic Imaging and Interventional Radiology, Pomeranian Medical University, Rybacka 1, 70204 Szczecin, Poland; michalgebkamed@gmail.com (M.G.); wojciech.poncyljusz@pum.edu.pl (W.P.); 2 Department of Neurology, Pomeranian Medical University, Rybacka 1, 70204 Szczecin, Poland; bajeranka1973@gmail.com (A.B.-C.); sandrapyza@gmail.com (S.P.); 3 Department of Biochemistry and Medical Chemistry, Pomeranian Medical University, Rybacka 1, 70204 Szczecin, Poland; chrissaf@mp.pl

**Keywords:** acute stroke, collateral circulation, spiral computed tomography, cerebral angiography, thrombectomy, artificial intelligence

## Abstract

Introduction: The aim of the study was to assess the impact of collaterals on the evolution of hypodensity on non-contrast CT (NCCT) in anterior circulation stroke with reperfusion by mechanical thrombectomy (MT). Methods: We retrospectively included stroke patients with middle cerebral artery occlusion who were reperfused by MT in early and late time window. Artificial intelligence (AI)-based software was used to calculate of hypodensity volumes at baseline NCCT (V1) and at follow-up NCCT 24 h after MT (V2), along with the difference between the two volumes (V2-V1) and the follow-up (V2)/baseline (V1) volume ratio (V2/V1). The same software was used to classify collateral status by using a 4-point scale where the score of zero indicated no collaterals and the score of three represented contrast filling of all collaterals. The volumetric values were correlated with the collateral scores. Results: Collateral scores had significant negative correlation with V1 (*p* = 0.035), V2, V2− V1 and V2/V1 (*p* < 0.001). In cases with collateral score = 3, V2 was significantly smaller or absent compared to V1; in those with collateral score 2, V2 was slightly larger than V1, and in those with scores 1 and 0 V2 was significantly larger than V1. These relationships were observed in both early and late time windows. Conclusions: The collateral status determined the evolution of the baseline hypodensity on NCCT in patients with anterior circulation stroke who had MT reperfusion. Damage can be stable or reversible in patients with good collaterals while in those with poor collaterals tissues that initially appear normal will frequently appear as necrotic after 24 h. With good collaterals, it is stable or can be reversible while with poor collaterals, normal looking tissue frequently appears as necrotic in follow-up exam. Hence, acute hypodensity represents different states of the ischemic brain parenchyma.

## 1. Introduction

The introduction of mechanical thrombectomy (MT) revolutionized the treatment of acute ischemic stroke in patients with large vessel occlusion (LVO).

Studies analysing the efficacy of MT have identified several pretreatment and treatment-related determinants of outcome. These include age, baseline National Institute of Health stroke scale (NIHSS), Alberta Stroke Program early CT score (ASPECTS), non-viable and salvageable tissue volume, time from stroke to reperfusion, degree of reperfusion, collaterals and hyperglycaemia [1,2,3,4,5,6].

Current guidelines state that the decision for MT should be guided by estimation of non-viable (core) and possibly-salvageable (penumbra) tissue volumes, especially in extended time windows of >6 h from the onset of stroke [7,8]. CT perfusion (CTP) is widely used to identify the predicted infarct core and penumbra volumes [8,9].

However, a recent study by Hendrix et al. [10] as well as the multicentre international CLEAR trial (published by Nguyen et al. in JAMA Neurology) [11] have shown that simple non-contrast CT (NCCT) alone may be just as useful as advanced imaging in selecting stroke patients with late-presenting LVO for MT, which could allow for a more pragmatic selection of patients in the extended window.

Although promising, such a simplified approach remains debatable because the importance of the acute hypodensity volume represented by the ASPECTS remains unclear and there is as yet no unequivocal evidence for how it should be interpreted. Additionally, it is not yet clear whether acute hypodensity at NCCT represents an irreversibly lost necrotic zone or salvageable tissue or some kind of mixture. These factors motivated us to design a study comparing baseline and follow-up hypodensities on NCCT and analysing their association with clinical and imaging parameters. We considered the factors which are thought to modify the evolution of hypodensity volume and focused on the status of the collateral circulation.

We used artificial intelligence (AI)—based software to assess the collaterals, and we used the same software package for calculation of the hypodensity volumes with revision by an experienced neuroradiologist. The rationale for this approach is the increasing ubiquity of AI-based software packages supporting physicians in the diagnostic work-up of stroke patients and the paucity of studies validating their accuracy in the assessment of collateral status.

The main aim of the study was to assess the evolution of hypodensity volumes on NCCT as correlated with collateral status in anterior circulation stroke patients successfully reperfused with MT.

## 2. Material and Methods

### 2.1. Study Participants and Design

The local Ethics Committee of the Pomeranian Medical University in Szczecin approved the study. Informed consent was waived considering the retrospective character of the study and the use of routinely performed procedures. Data of consecutive patients who presented to our comprehensive stroke centre from February 2019 to October 2021 with suspected acute stroke and underwent head NCCT were retrospectively retrieved using our Radiological Information System and Picture Archiving and Communication System. The initial inclusion criteria were NIHSS score ≧ 6 and modified Rankin Scale (mRS) score ≦ 2. Patients with intracranial haemorrhage or ASPECTS score < 6 at baseline NCCT were excluded. Those within the therapeutic window of ≦4.5 h without contraindications received intravenous thrombolysis (IVT).

The subsequent parts of diagnostic work-up included CTP and CT angiography (CTA), and MT was indicated in patients with LVO detected at CTA within a therapeutic window of ≦6 h. Patients in extended therapeutic windows of >6 h were evaluated for MT according to DEFUS-3 or DAWN criteria [12,13]. Patients with wake-up stroke or unknown time of stroke onset were excluded because time from the onset of stroke to MT was required for further analysis. Follow-up NCCT was performed about 24 h after MT.

We included patients with the middle cerebral artery (MCA) LVO because the AI-based software performs calculations only in the territory supplied by this vessel.

Another inclusion criterion was MT with cerebral infarction scale (TICI) 2b or 3 reperfusion. Patients with TICI 0 to 2a reperfusion were not analysed to exclude any confounding effects because gradual tissue loss probably continues beyond 24 h in cases of poor reperfusion and the hypodensity volume at the follow-up NCCT 24 h post MT may not represent the final infarct volume. Another reason was inhomogeneity of this population as TICI 0/1 means no or minimal reperfusion while in TICI 2a partial reperfusion reaching almost 50% of ischaemic territory can be achieved. This difference could interfere with assessment of the impact of collaterals. Additionally, the excluded group including only 58 patients was too small for performing reliable statistical analysis and drawing general conclusions. Besides, we were focused on investigating whether baseline hypodensity would progress, resolve or stabilize after nearly complete or complete reperfusion with TICI 2B or 3 depending on collateral status.

**Figure 1:**
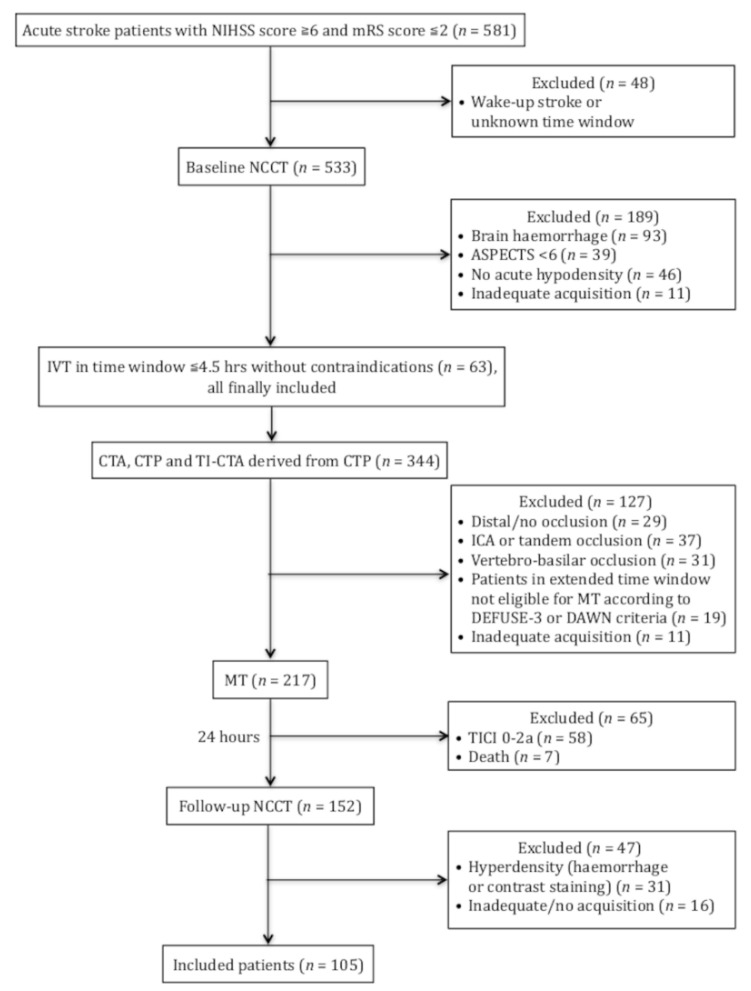
Flow chart of the study. NIHSS—National Institute of Health stroke scale; mRS—modified Rankin scale; IVT—intravenous thrombolysis; ASPECTS—Alberta Stroke Program early CT score; MT—mechanical thrombectomy; NCCT—non-contrast CT; TICI—thrombolysis in cerebral infarction scale; CTA—CT angiography; CTP—CT perfusion; TI-CTA—timing invariant CT angiography; ICA—internal carotid artery.

The remaining exclusion criteria were lack of a 24-h follow-up NCCT after MT, any hyperdensity representing haemorrhage or contrast staining on follow-up NCCT and technical inadequacy of any CT examination. We also excluded patients without acute hypodensity on baseline NCCT because in these cases it was impossible to calculate V2/V1. The study selection flow chart is presented in Figure 1.

### 2.2. Image Acquisition and Reconstruction

All patients were scanned on a 128-slice multi-detector CT SOMATOM Definition AS+ (Siemens Healthcare GmbH, Erlangen, Germany).

NCCT, CTP and CTA were performed with the technique and parameters described in previous study [14].

### 2.3. Image Analysis

A board certified interventional neuroradiologist with 15 years of experience (M.S.) evaluated all CTAs and recorded the presence, side, and location of LVO. He was blinded to all clinical and imaging data.

All CTP exams were assessed on a regular basis by the radiologist on duty for clinical use, but those results were not included in this analysis.

The baseline and follow-up NCCT hypodensity volumes were calculated with the automated e-ASPECTS tool, a part of the e-Stroke Cloud imaging suite (version 10.1p4, Brainomix Ltd., Oxford, UK).

The collateral status was scored by using the e-Stroke Cloud e-CTA tool.

The e-Stroke Cloud image processing algorithms follow an AI approach, with a combination of traditional 3D graphics and statistical methods and machine learning classification techniques. The input DICOM data are first resampled to correct any gantry tilt and standardize the input resolution. Then, a fast proprietary registration approach is applied to re-align the data, removing any tilt and rotation. This ensures that the image is presented in a standard reference frame, which simplifies human interpretation of the scan.

To identify acute hypodensity volumes e-ASPECTS tool compares corresponding regions of normal and ischaemic hemisphere voxel by voxel. Acute hypodensity is defined by difference between hemispheres in Hounsfield Units with threshold values. The process is based on AI combined with 3D graphics and statistical methods. To eliminate the possibility that some subacute changes on the follow-up NCCT were missed by the software all follow-up exams were revised by interventional neuroradiologist (M.S.). Manual correction by radiologist was needed in 6 cases.

The e-CTA process uses a combination of machine learning and deep learning algorithms to identify LVO in the anterior circulation.

The e-CTA also uses multiphase images derived from CTP to define and quantify asymmetrical contrast densities from which the degree of collateral blood vessel density can be estimated, quantified both as a relative percentage and using the Tan collateral score [15].

These algorithms have been trained on a large dataset (>10,000 images) containing a wide range of real-world CT scans from stroke patients and negative controls as well as ground-truth data from additional imaging data such as magnetic resonance (MR) images acquired within 1 to 2 h of the CT scan along with other modalities and clinical information. The training dataset contains examples of CT scans captured with scanners from all major manufacturers and from a wide range of countries worldwide. The e-Stroke Cloud suite received its European Union CE mark certification in 2018.

For collateral scoring, CTA images (axial, coronal and sagittal maximal intensity projections (MIPs) with 3-mm thickness) were automatically reconstructed from the CTP source data as timing-invariant (TI)-CTA using e-CTA. TI-CTA visualizes vessels by overlapping all timeframes and displaying maximal enhancement over time. This technique is timing invariant because of the choice of the temporal maximum, meaning that the peak enhancement of a vessel is displayed regardless of the delay of contrast arrival. Accurate assessment of the collaterals is possible because TI-CTA is not sensitive to delayed arrival of contrast material in cerebral vessels. This technique was introduced by Smit et al. [16]. It has been well-documented for collateral scoring [17,18,19,20], and a recent study by Nail et al., has confirmed its superiority in the assessment of collaterals compared to single phase CTA [21].

As a reference, collateral scoring was also assessed by a board certified interventional neuroradiologist (M.S.) who analysed the same TI-CTA images derived from the CTP as were used by the e-CTA software. The level of interobserver agreement in collateral scoring between the neuroradiologist and e-CTA was calculated, and the collateral scores from the neuroradiologist were used as the reference for further analyses.

### 2.4. Data Collection

Beside age and sex, the following data were recorded: occlusion site, classified as the M1 or M2 segment of the MCA; technique and result of MT as TICI 2b or 3 based on the report in the radiological information system; time gap from the onset of stroke symptoms or last seen well to the arterial puncture preceding MT, grouped as ≤3 h, 3 to 6 h and >6 h, and the administration of IVT.

The following data were registered based on the e-ASPECT calculations for the NCCT:

- V1, defined as the volume of acute hypodensity at the baseline NCCT;
- V2, defined as volume of hypodensity at the 24-h follow-up NCCT;
- V2 − V1, defined as the difference between the follow-up and baseline hypodensity volumes;
- V2/V1, reflecting the follow-up/baseline volume ratio.

The results provided by the AI-based software were revised by the neuroradiologist (M.S.) to eliminate significant miscalculations.

The collateral scores from e-CTA and the neuroradiologist (M.S.) were recorded by using the scoring system proposed by Tan et al. [15]. This system classifies the collateral circulation status into 4 grades, as follows:

- 0, no contrast filling of the collateral supply to the occluded MCA territory;
- 1, collateral supply to >0% but ≤50% of the occluded MCA territory;
- 2, collateral supply to >50% but <100% of the occluded MCA territory;
- 3, contrast filling of the collateral supply to 100% of the occluded MCA territory.

The level of interobserver agreement in collateral scoring between the neuroradiologist and e-CTA was calculated, and the collateral scores from the neuroradiologist were used as the reference for further analyses.

The time gap from MT to follow-up NCCT was noted.

The processing time for e-Stroke, defined as the time from data transmission to the reception of results, was also recorded.

### 2.5. Statistical Analysis

Continuous variables were reported as mean (standard deviation, SD) or median (interquartile range, IQR) and were compared with the Student’s *t* test, one-way analysis of variance, Mann–Whitney, or Kruskal–Wallis tests as appropriate. Categorical variables were compared with the χ^2^ or Fisher exact tests as appropriate. Spearman’s rank correlation coefficient (r) was used for calculation of correlation between continuous variables. The level of interobserver agreement in collateral scoring between the neuroradiologist and the e-CTA was assessed with Krippendorff’s alpha coefficient.

Statistical calculations were performed by a biostatistician (K.S.) using Statistica 13 data analysis software (TIBCO Software Inc., Palo Alto, CA, USA) with Plus Bundle 5.0.96.

## 3. Results

The data of 581 patients were collected, and after implementation of the exclusion criteria, 105 were finally included in the study.

There were 53 women and 52 men with a mean age of 69.7 years (±11.6 years; range 36 to 92 years). Detailed patient characteristics are presented in Table 1.

The mean time gap from the onset of stroke symptoms or last seen well to the arterial puncture preceding MT was 4 h 12 min (range 1 h 36 min to 9 h 40 min). Sixty-six (62.9%) patients were within the therapeutic window of 4.5 h for IVT, and IVT was administered in all of them except three who had contraindications. V1 was significantly smaller in the patients who had IVT compared to those who did not (mean 14.7 mL, ±16.2 mL vs. mean 19.3 mL, ±15.6 mL; *p* = 0.04).

For MT, an aspiration technique alone was used in 69 (65.7%) patients, aspiration with stent retriever in 31 (29.5%) and stent retriever alone in 5 (4.8%).

The mean time to the follow-up NCCT after MT was 24 h 33 min (range, 22 h 16 min to 27 h 32 min).

The collateral score was significantly negatively correlated with all hypodensity volume parameters, although the correlation with V1 was borderline (r = −0.206; *p* = 0.035). The strongest correlation was found with V2 (r = −0.722; *p* < 0.001). Collateral score significantly negatively correlated with V2–V1 (r = −0.642; *p* < 0.001) and V2/V1 (r = −0.646; *p* < 0.001).

Analysis performed separately for different times to treatment (≤3 h, 3–6 h and >6 h) revealed the same associations between collateral status and hypodensity volume parameters as for the entire study population. These findings are presented in Table 2 and Table 3.

No correlation was found between collateral score and other parameters.

**Figure 2:**
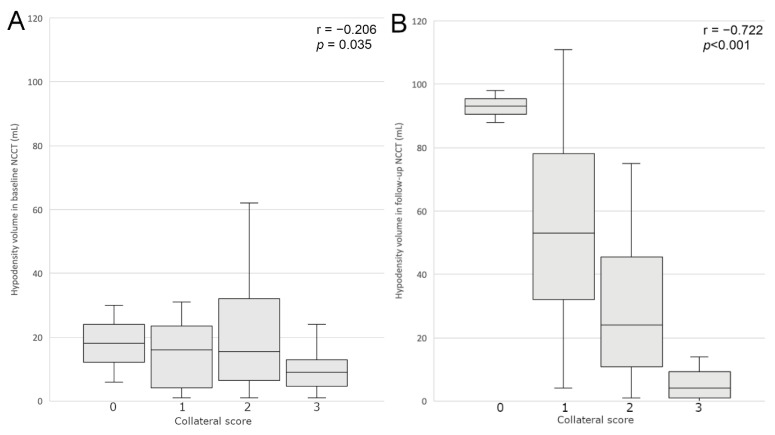
Correlation of baseline (**A**) and follow-up (**B**) hypodensity in NCCT with collateral status.

**Figure 3:**
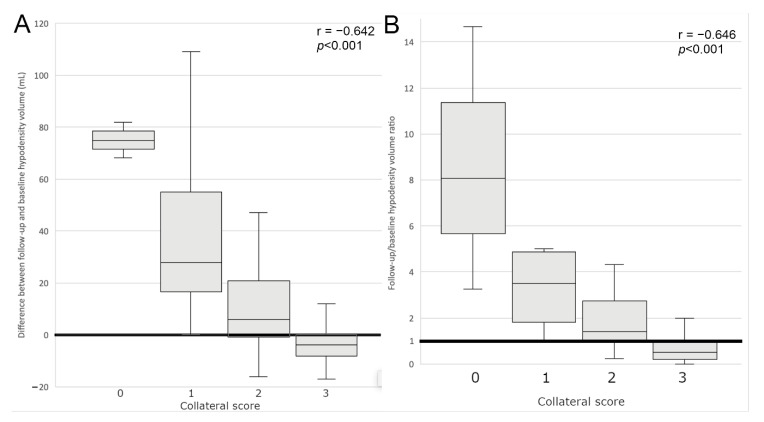
Correlation of hypodensity change (**A**) and follow-up/baseline hypodensity ratio (**B**) in NCCT with collateral status.

**Figure 4:**
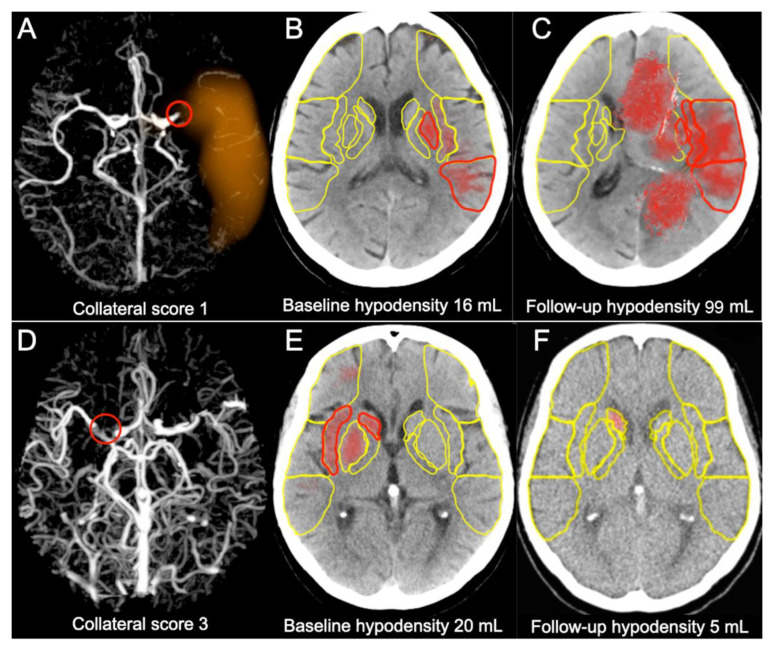
Representative cases with poor and good collaterals. Upper row presents the case of 79 y/o female treated 2 h and 43 min from onset of stroke with TICI 3. IVT was not administered due to the usage of oral anticoagulants: (**A**) left M1-MCA occlusion shown by red circle on timing-invariant CTA and poor collaterals scored as 1 by e-CTA; (**B**) e-ASPECTS assessed acute hypodensity volume for 16 mL on the baseline NCCT (red areas); (**C**) the follow-up NCCT revealed significant increase of hypodensity calculated for 99 mL by e-ASPECTS (red areas). Lower row presents the case of 72 y/o female treated 4 h and 50 min from onset of stroke with TICI 3: (**D**) right M1-MCA occlusion shown by red circle on timing-invariant CTA and good collaterals scored as three by e-CTA; (**E**) e-ASPECTS assessed acute hypodensity volume for 20 mL on the baseline NCCT (red areas); (**F**) the follow-up NCCT revealed significant decrease of hypodensity calculated for 5 mL by e-ASPECTS (red area). Regions used for calculation of ASPECTS are marked in yellow.

In the group with collateral Grade 3 the follow-up hypodensity volume appeared to be smaller than the baseline volume, and was absent in some cases. In the group with collateral Grade 2 there was a slight increase of the hypodensity volume and in the groups with collateral Grades 0 and 1 the increase was significant, as presented in Figure 2 and Figure 3. Representative cases are shown in Figure 4.

**Figure 5:**
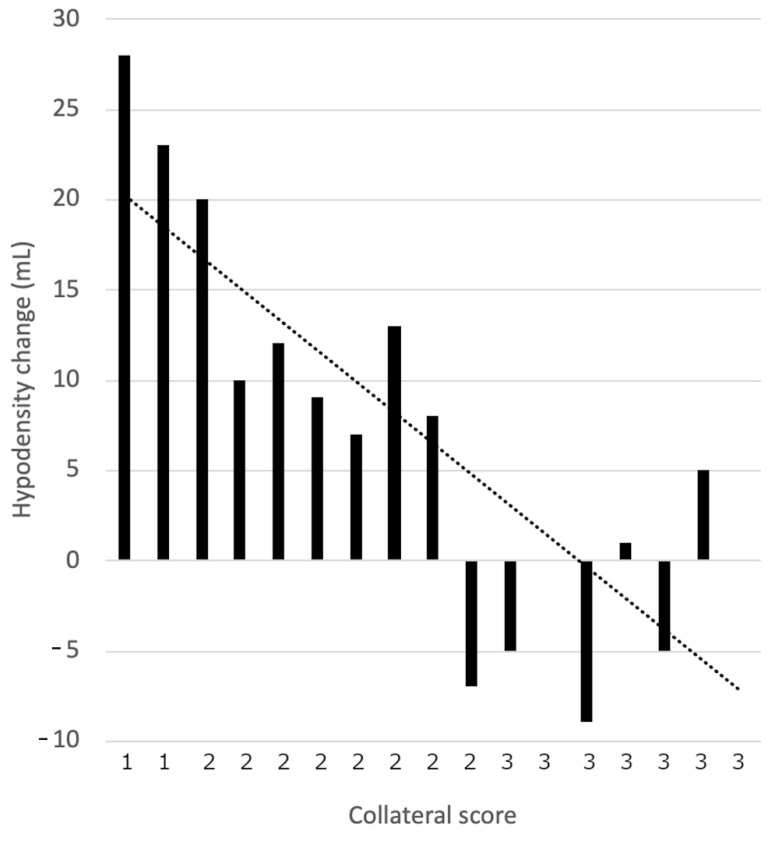
Correlation of hypodensity change with collateral status in patients treated in late time window of >6 h. The results for each particular collateral score are presented according to the date of treatment.

There were 17 patients (17/105; 16.2%) who were treated with MT in later time windows (time from onset of stroke to groin puncture ranging from 6 h 10 min to 9 h 40 min). Among seven who had collateral scores of three, three showed hypodensity volume decrease; two showed no change and two showed increases. The collateral scores were 1 or 2 in the remaining ten cases, and only one of these, a patient with score two, showed decreased hypodensity. Volume increases were observed in the other nine and the greatest volume increases were observed in two cases with collateral score one. These results are presented in Figure 5.

The level of interobserver agreement in collateral scoring between the neuroradiologist and the e-CTA algorithm was very good (Krippendorff’s alpha = 0.83).

The mean processing time was 103 s (±28 s) for e-ASPECTS and 81 s (±23 s) for e-CTA.

## 4. Discussion

Our study showed that the collateral circulation status in stroke impacts the baseline hypodensity volume at NCCT and modifies its evolution after recanalization. These effects are independent of time from onset of stroke to groin puncture or IVT administration.

Patients who were eligible for and received IVT had moderately smaller baseline hypodensity volumes compared to those without thrombolysis probably because IVT administration is avoided in patients with large hypodensities at baseline NCCT due to the high risk of haemorrhagic transformation.

Although we observed significant correlation between collateral scores treated as rank variables and baseline hypodensity volumes (*p* = 0.035), we did not find marked differences in baseline volumes between collateral scores treated as unordered levels of nominal variables. This relationship was completely different in the follow-up exams. In patients with the best collateral status (score three), the follow-up NCCT usually showed partial or even complete regression of hypodensity. In the group with decent collateral score of two stabilization or slight increase of hypodensity was typically observed. In patients with poor or absent collaterals (scores one and zero) significant progression of hypodensity was detected despite efficacious recanalization. These dependencies were observed in both early and in late time windows.

Although our study was limited to assessment of a single imaging factor as an outcome measure, the findings could be regarded as consistent with results of studies showing that collaterals determine functional outcome by the extensively used mRS at 90 days [22,23,24,25,26,27,28,29,30,31,32].

A novelty of our study is the application of AI-based software instead of conventionally used human reading in the assessment of collaterals.

Our study also adds evidence that baseline NCCT does not reflect the true status of ischemic brain parenchyma.

We showed that baseline hypodensity on NCCT can be reversible in patients with good collaterals. On the contrary, in the presence of poor collaterals, tissue that looked normal at the baseline NCCT frequently progressed to necrotic at the follow-up exam despite successful recanalization. Moreover, it appears that such evolution is independent of the time from stroke to reperfusion, as it was seen in cases with of both early and extended therapeutic windows. This may lead to the interpretation that the initial pattern on NCCT represents different states of ischaemic brain tissue. With good collaterals, acute hypodensity is not consistent with necrotic zone as it is potentially salvageable after reperfusion. Contrarily, with poor collaterals, initially normal looking tissue could be already irreversibly damaged before reperfusion. In such cases this zone, which looks normal at the baseline NCCT appears as necrotic at the follow-up exam. This phenomenon could be explained by a well-known fact, that NCCT detects the signs of acute ischaemia with some delay.

This puts into question the reliability of ASPECTS as an independent predictor of imaging and clinical outcomes because it directly represents the acute hypodensity volume, and there may be some implications regarding the weight of the ASPECTS score in the selection of stroke patients for MT.

According to current guidelines [7,8], in the early time window, MT should be performed in all patients with ASPECTS ≥ 6 and MT is generally not recommended below this threshold and can only be performed under certain circumstances. For using MT in late time windows, ASPECTS ≥ 6 is obligatory in association with advanced imaging confirming core, penumbra and mismatch regions which fulfil DEFUSE-3 or DAWN criteria [12,13].

However, advanced imaging can be prohibitively expensive and is not widely available across the world, and requiring this technology for selection excludes many patients from being considered for late MT.

This issue has been addressed in two recent studies by Hendrix et al. [10] and Nguyen et al. [11] which showed that there were no significant differences in clinical outcomes in proximal anterior stroke treated with MT in the late time window between patients selected solely with simple NCCT and those selected with advanced imaging. The authors concluded that their findings have the potential to widen the indication for treating patients in the extended window using simpler, less costly, and easier to implement NCCT imaging.

Such an approach seems to be promising. Although our results show that hypodensity at baseline NCCT may be an unreliable indicator of final infarct in many cases because it can significantly increase or decrease depending on collateral status, the proposed simplified selection criteria could be optimized by including the scoring of collaterals which is a strong modifier of acute hypodensity in NCCT and can be assessed with routinely performed CTA without requirement for advanced imaging techniques.

Without doubt, this hypothesis needs to be verified by further comprehensive research.

### Study Limitations

The study population was limited to stroke patients with M1 and M2-MCA occlusions treated with MT resulting in TICI 2b and 3. We did not include untreated patients, those with tandem or ICA occlusions or those with TICI 0 to 2a. We also did not analyse all known modifiers of outcome and there was no analysis of functional outcomes. Another limitation is the use of only one type of CT scanner. Comparing results from different scanners would be needed to verify that the trend found in this study holds true for other acquisition parameters. Lastly, the population with collateral score zero consisted of only three patients and only 17 patients were treated in the late window, which could bias the results.

## 5. Conclusions

In anterior circulation stroke patients who were reperfused with MT, evolution of hypodensity volumes on NCCT was correlated with collateral status in both early and late time windows. Baseline hypodensity is commonly stable or can be reversible with good collaterals, while with poor collaterals, normal looking tissue frequently appears as necrotic in follow-up exam despite apparently successful recanalization.

Considering that acute hypodensity represents different states of ischemic brain parenchyma, ASPECTS as predictor of outcome should be analysed in association with collateral status.

## Figures and Tables

**Table 1:** Demographic and clinical variables according to collateral status.

Parameter	Collateral Score 0 *n =* 3	Collateral Score 1 *n =* 14	Collateral Score 2 *n =* 48	Collateral Score 3 *n =* 40	*p* Value
Age, years, mean (range)	76 (64–89)	73 (43–90)	69 (40–84)	69 (36–92)	0.753
Females, *n* (%)	2 (66.7)	7 (50)	21 (43.8)	23 (57.5)	0.631
TICI score, *n* (%)					0.181
2b	0 (0)	2 (14.3)	15 (31.3)	9 (22.5)	—
3	3 (100)	12 (85.7)	33 (68.7)	31 (77.5)	—
Occlusion localization, *n* (%)					0.112
M1-MCA	3 (100)	9 (64.3)	34 (70.8)	29 (72.5)	—
M2-MCA	0 (0)	5 (35.7)	14 (29.2)	11 (27.5)	—
Time from stroke onset to groin puncture, *n* (%)					0.193
≤3 s	0 (0)	5 (35.7)	20 (41.7)	13 (32.5)	—
3–6 h	3 (100)	7 (50)	20 (41.7)	20 (50)	—
>6 h	0 (0)	2 (14.3)	8 (16.6)	7 (17.5)	—
IVT, *n* (%)	2 (66.7)	9 (64.3)	29 (60.4)	23 (57.5)	0.546
V1, mL, median (IQR)	18 (12–24)	16 (4–23.5)	15.5 (6.5–32)	9 (4.8–13)	0.039
V2, mL, median (IQR)	93 (90.5–95.5)	53 (32–78)	24 (10.8–45.5)	4 (1–9.3)	<0.001
V2− V1, mL, median (IQR)	75 (71.5–78.5)	28 (16.5–55)	6 (0.8–20.8)	−4 (−8–0)	<0.001
V2/V1	8.1 (5.7–11.4)	5 (1.8–4.9)	1.4 (1–2.7)	0.5 (0.2–1)	<0.001

IVT—intravenous thrombolysis; M1-MCA—M1 segment of the middle cerebral artery; M2-MCA—M2 segment of the middle cerebral artery; TICI—thrombolysis in cerebral infarction scale; V1—volume of acute hypodensity in baseline NCCT; V2—volume of hypodensity in follow-up NCCT; V2 − V1—difference between follow-up and baseline volume of hypodensity; V2/V1—follow-up/baseline volume ratio.

**Table 2:** Correlation between collateral score and hypodensity volume parameters depending on the time to treatment.

Parameter	≤3 h * *n* = 38		3–6 h * *n =* 50		>6 h * *n =* 17	
	r	*p* Value	r	*p* Value	r	*p* Value
V1	−0.270	0.011	−0.210	0.033	−0.505	<0.001
V2	−0.601	<0.001	−0.624	<0.001	−0.740	<0.001
V2− V1	−0.580	<0.001	−0.646	<0.001	−0.824	<0.001
V2/V1	− 0.289	0.006	− 0.361	<0.001	− 0.569	<0.001

V1—volume of acute hypodensity in baseline NCCT; V2—volume of hypodensity in follow-up NCCT; V2–V1—difference between follow-up and baseline volume of hypodensity; V2/V1—follow-up/baseline volume ratio; r—Spearman’s rank correlation coefficient; *—the time gap from the onset of stroke symptoms to the arterial puncture.

**Table 3:** Distribution of hypodensity volumetric parameters for different collateral scores depending on the time to treatment.

Parameter	Collateral Score 0	Collateral Score 1	Collateral Score 2	Collateral Score 3	*p* Value
≤3 h * (*n* = 38)					
V1, mL, median (IQR)	ND	16 (16–22)	16.5 (7.3–29.5)	9 (8–12)	0.041
V2, mL, median (IQR)	ND	76 (74–80)	24.5 (9.5–34.8)	3 (1–5)	<0.001
V2− V1, mL, median (IQR)	ND	60 (49–64)	0.5 (−1–21)	−8 (−9–−3)	<0.001
V2/V1	ND	4 (3.5–4.8)	1 (0.9–2.1)	0.3 (0.1–0.4)	<0.001
3–6 h * (*n =* 50)					
V1, mL, median (IQR)	6 (5–18)	9 (2.5–24)	11 (3.8–29.3)	9 (3.5–14.8)	0.062
V2, mL, median (IQR)	88 (46–93)	42 (33.5–55.5)	22 (12.3–39)	3.5 (1.8–10.3)	<0.001
V2− V1, mL, median (IQR)	68 (34–75)	27 (16.5–41.5)	7 (0.5–24.3)	−3 (−7–0)	<0.001
V2/V1	3.3 (2.1–9)	4.7 (1.8–17.8)	1.7 (1–5.4)	0.5 (0.4–1)	<0.001
>6 h * (*n =* 17)					
V1, mL, median (IQR)	ND	39.5 (25.3–53.8)	34.5 (13.8–43)	9 (5.5–11.5)	0.039
V2, mL, median (IQR)	ND	65 (49.5–80.5)	44.5 (23.5–55.5)	10 (4.5–11.5)	<0.001
V2− V1, mL, median (IQR)	ND	25.5 (24.3–26.8)	10 (7.8–12.3)	0 (−5–0.5)	<0.001
V2/V1	ND	2.3 (1.8–2.7)	1.4 (1.2–1.9)	1 (0.5–1.1)	<0.001

V1—volume of acute hypodensity in baseline NCCT; V2—volume of hypodensity in follow-up NCCT; V2–V1—difference between follow-up and baseline volume of hypodensity; V2/V1—follow-up/baseline volume ratio; *—the time gap from the onset of stroke symptoms to the arterial puncture.

## Data Availability

Mendeley Data Repository at https://data.mendeley.com (accessed on 25 November 2021). Dataset name: Sawicki, Marcin (2022), “Evolution of Hypodensity on Non-contrast CT in Correlation with Collaterals in Anterior Circulation Stroke with Successful Endovascular Reperfusion”, Mendeley Data, V1, doi:10.17632/vc3cyv4tsc.1
